# Focal Recurrent Copy Number Alterations Characterize Disease Relapse in High Grade Serous Ovarian Cancer Patients with Good Clinical Prognosis: A Pilot Study

**DOI:** 10.3390/genes10090678

**Published:** 2019-09-05

**Authors:** Matteo Dugo, Andrea Devecchi, Loris De Cecco, Erika Cecchin, Delia Mezzanzanica, Marialuisa Sensi, Marina Bagnoli

**Affiliations:** 1 Platform of Integrated Biology, Department of Applied Research and Technological Development, Fondazione IRCCS Istituto Nazionale dei Tumori, 20133 Milan, Italy (A.D.) (L.D.C.) (M.S.); 2 Experimental and Clinical Pharmacology Unit, Centro di Riferimento Oncologico, IRCCS National Cancer Institute, 33081 Aviano, Pordenone, Italy; 3 Molecular Therapy Unit, Department of Research, Fondazione IRCCS Istituto Nazionale dei Tumori, 20133 Milan, Italy

**Keywords:** ovarian cancer, platinum resistance, focal copy number alterations, whole exome sequencing

## Abstract

High grade serous ovarian cancer (HGSOC) retains high molecular heterogeneity and genomic instability, which currently limit the treatment opportunities. HGSOC patients receiving complete cytoreduction (R0) at primary surgery and platinum-based therapy may unevenly experience early disease relapse, in spite of their clinically favorable prognosis. To identify distinctive traits of the genomic landscape guiding tumor progression, we focused on the R0 patients of The Cancer Genome Atlas (TCGA) ovarian serous cystadenocarcinoma (TCGA-OV) dataset and classified them according to their time to relapse (TTR) from surgery. We included in the study two groups of R0-TCGA patients experiencing substantially different outcome: Resistant (R; TTR ≤ 12 months; n = 11) and frankly Sensitive (fS; TTR ≥ 24 months; n = 16). We performed an integrated clinical, RNA-Sequencing, exome and somatic copy number alteration (sCNA) data analysis. No significant differences in mutational landscape were detected, although the lack of BRCA-related mutational signature characterized the R group. Focal sCNA analysis showed a higher frequency of amplification in R group and deletions in fS group respectively, involving cytobands not commonly detected by recurrent sCNA analysis. Functional analysis of focal sCNA with a concordantly altered gene expression identified in R group a gain in Notch, and interferon signaling and fatty acid metabolism. We are aware of the constraints related to the low number of OC cases analyzed. It is worth noting, however, that the sCNA identified in this exploratory analysis and characterizing Pt-resistance are novel, deserving validation in a wider cohort of patients achieving complete surgical debulking.

## 1. Introduction

High grade serous ovarian cancer (HGSOC) is the most common and lethal epithelial ovarian cancer (EOC) subtype, causing 70–80% of ovarian cancer deaths worldwide [1]. Due to the lack of specific symptoms it is generally diagnosed at advanced stages when it has diffusely metastasized into the peritoneal cavity. Standard treatment includes aggressive primary debulking surgery (PDS) followed by platinum (Pt)-based therapy; but, despite the improvement of surgical approaches and drug development, survival rate has changed little in the last decades [2].

Pt-based therapy remains the cornerstone treatment type and, currently, *BRCA1/2* mutation status is the only biomarker that allows up-front identification of patients with Pt-sensitive or resistant disease [3]. As a consequence, around 30% of patients undergoing Pt-based chemotherapy do not respond to treatment. Also, around 80% of those patients achieving complete response will relapse with a median progression-free survival of 18 months, developing a disease that progressively becomes Pt-resistant, a largely incurable state [2,3].

The opportunity to effectively treat and control HGSOC progression is limited by tumor heterogeneity and genomic instability. HGSOC following p53 mutation undergo multiple sequential mutational processes that shape a complex genome, strongly dominated by somatic copy number aberrations (sCNA). As a result, HGSOC like other CNA driven tumors, as esophageal cancer, non-small-cell lung cancer and triple negative breast cancer, have a low frequency of recurrent oncogenic mutations and a few recurrent sCNA [4]. These multiple mutational forces acting on HGSOC cause difficulties in the identification of targetable genetic lesion(s).

At present, no residual tumor (R0) after PDS is the most important prognostic factor for survival in advanced stage disease [2]. Analyzing clinical data of The Cancer Genome Atlas (TCGA) ovarian serous cystadenocarcinoma (TCGA-OV) we observed that in the group of patients experiencing early relapse were included also those who received optimal clinical treatment (Pt-based therapy and no residual disease after PDS) supporting the notion that intrinsic characteristic(s) of the tumor play a major role in the lack of responsiveness.

The aim of the present pilot study is to decipher the genomic landscape characterizing the highly selected cohort of HGSOC patients who experienced an early relapse, in spite of their expected favorable outcome as assessed by clinical parameters.

## 2. Materials and Methods

### 2.1. Data Source and Samples Selection

Mutational and copy number data of TCGA-OV samples were downloaded from the Broad Institute Firehose web portal (https://gdac.broadinstitute.org/) with data version 2016_01_28. Clinical data were obtained from the ovarian cancer landmark paper [5]. RNA-Seq raw counts data were obtained from the Genomic Data Commons data portal (https://portal.gdc.cancer.gov/) with accession date 12th March 2019.

For genomic analyses we selected patients with: (i) no residual disease (R0) after PDS; (ii) whole-exome sequencing data available; (iii) sCNA data available; (iv) a follow-up time ≥ 12 months. Forty-eight patients having these characteristics were then classified according to their time to relapse (TTR). Since the time of end-of-treatment was not recorded, the disease-free interval was calculated from the date of surgery. Patients were categorized on the basis of disease-free period length and we identified two subgroups having very different TTR: the refractory/resistant (R) group with TTR ≤ 12 months (n = 11), and the frankly Sensitive (fS) groups with TTR ≥ 24 months (n = 16). These 27 patients (5.9% of the entire TCGA-OV cohort) constitute the TCGA-OV27 cohort, analyzed in the present study. All analyses described in the following sections were performed in the R environment version 3.5.2.

### 2.2. RNA-Seq Data Analysis

RNA-Seq data were available for 23 patients (9 R and 14 fS) of the TCGA-OV27 dataset. Raw read counts were normalized using the Trimmed Mean of M-values (TMM) method [6], implemented in the edgeR Bioconductor package [7]. TMM estimates a scaling factor used to reduce technical bias between samples due to differences in library size. Normalized data were then filtered removing genes with at least 1 count per million reads in less than 5% of samples. The final dataset included 23391 unique genes. Differential expression between R and fS was performed using the limma/voom pipeline [8]. *p*-values were corrected for multiple testing using the Benjamini–Hochberg false discovery rate (FDR) method. Ensembl gene IDs were associated to HUGO gene symbols using the GENCODE v22 annotation. Gene Set Enrichment Analysis [9] between R and fS was performed using the Fast Gene Set Enrichment Analysis (fgsea) package ranking genes according to the t-statistic obtained with limma. Gene sets of the “Hallmark” collection from the Molecular Signatures Database (MSigDB, http://software.broadinstitute.org/gsea/msigdb/) were tested. Gene sets with an FDR < 0.05 were considered significant.

### 2.3. Mutational Data Analysis

Mutation Annotation Format (MAF) files used to store somatic variants detected were summarized, analyzed, annotated, and visualized using the maftools Bioconductor package [10]. Only variants assumed to have high or moderate (disruptive) impact in the protein, probably causing protein truncation, loss of function or triggering nonsense mediated decay were included in the analysis of most frequently mutated genes. For the calculation of tumor mutational load we considered both high/moderate impact mutation and all somatic mutations.

The DeconstructSigs package [11] was used to perform the mutational signature analysis. This tool evaluates the contribution of 30 signatures reported in COSMIC (https://cancer.sanger.ac.uk/cosmic/signatures) [12] to the mutational profile of each sample. Mutational signatures were calculated considering all somatic mutations in a given sample. The obtained signature scores were then analyzed in association with sensitivity class using Wilcoxon rank-sum test. Samples were grouped according to the top-5 most contributing mutational signatures using unsupervised hierarchical clustering performed with Euclidean distance and Ward linkage.

To identify mutations associated to sensitivity class we used the clinicalEnrichment function of *maftools* package [10] that performs Fisher’s exact tests to identify mutated genes associated with the class of interest. Analysis at the level of oncogenic pathways described in Sanchez-Vega et al. [13] was performed using the *OncogenicPathways* function of *maftools*. For each sample we classified each pathway as mutated if at least one of its genes carried a mutation. We then associated mutated pathways to sensitivity class using Fisher’s exact test. The same analysis was repeated using the “Hallmark” gene sets from MSigDB.

### 2.4. sCNA Data Analysis

Genomic Identification of Significant Targets in Cancer (GISTIC) [14] algorithm was used to analyze sCNA data.

Segmented copy number data were analyzed using GISTIC [14] to identify significantly recurrent sCNA in the whole TCGA-OV27 cohort, independently of sensitivity class. GISTIC output was parsed using the maftools package [10]. In addition to the regions recurrently affected by sCNA, GISTIC provides a gene-level copy number status for all genes of the genome in each sample (all_thresholded.by_genes.txt output file). Thus, we tested the association with sensitivity class both for recurrently amplified or deleted regions (GISTIC FDR < 0.1) and for each single gene. For these analyses amplifications and deletions were analyzed separately. For amplifications, a region was assigned a value of 1 if amplified or 0 if the region was not altered or deleted. The same criterion was applied to deletions. The binary amplification and deletion data were then analyzed in relation to sensitivity class using Fisher’s exact test. *p*-values were corrected for multiple testing using the Benjamini-Hochberg FDR method.

Per sample genomic instability was calculated according to: (i) The number of segments in the segmented copy number data; (ii) the total number of genes with a copy number alteration; (iii) the sum of deleted or amplified genes. Association between genomic instability and sensitivity class was assessed by Wilcoxon rank-sum test.

### 2.5. Statistical Power and Sample Size Calculation

The statistical power for Fisher’s exact test applied to the TCGA-OV27 cohort for mutational and sCNA data analyses was calculated using the power2x2 function of the exact2x2 R package. From TCGA-OV27 data we observed that the genes mostly associated to the phenotype of interest were altered (mutated, amplified or deleted) in 27% and 94% of R and fS patients, respectively. Considering these proportions and hypothesizing to test 20,000 genes, the present study has a statistical power of 2.4% of detecting at least one significant finding at an FDR threshold of 5%. To achieve a power of 80% at the same FDR threshold at least 31 patients per group are required. This sample size was calculated using the ss2x2 function of the R-package.

### 2.6. Integrated sCNA and RNA-Seq Functional Analysis

Functional analysis was carried out on a subset of genes that showed coherent copy number status and differential expression in R compared to fS patients. Over-representation of molecular and cellular functions in the list of selected genes was carried out using: (i) Reactome canonical pathways gene sets from the C2 collection of MSigDB (http://software.broadinstitute.org/gsea/msigdb/annotate.jsp) to map the genes in known functional pathways; (ii) Ingenuity^®^ Pathway Analysis (IPA^®^, Qiagen; Bioinformatics, Redwood City, CA, USA; http://www.qiagen.com/ingenuity) to derive predictions about the activation status. Enrichments with an FDR < 0.05 were considered statistically significant.

## 3. Results

From TCGA-OV dataset we selected patients with no residual disease (R0) after PDS with WES and sCNA data available and with a follow-up time ≥ 12 months.

Then, considering that the subgroup of R0 patients is expected to have a good prognosis, for the pilot analysis we further refined the cohort selecting Resistant (R, n = 11) with an unfavorable outcome and frankly Sensitive (fS, n = 16) patients. Overall 27 patients, the TCGA-OV27 cohort, were included in the study and their associated clinical data are summarized in Table 1.

Transcriptomic analysis of TCGA-OV27 cohort did not reveal differentially expressed genes between R and fS patients at an FDR < 0.05. However, when we considered a nominal *p*-value < 0.05 and a fold-change of 2, 210 and 214 genes were down- and up-regulated respectively, in R patients. Comparison of the two groups using GSEA highlighted 29 hallmark gene sets significantly enriched in one of the two groups at an FDR < 0.05. (Figure S1). In particular, 1 gene set related to oxidative phosphorylation was positively enriched in fS patients while the remaining 28 gene sets were positively enriched in the R group. Overall, gene sets positively enriched in R patients are supporting of a more aggressive phenotype for this subset of tumors but did not highlighted specific mechanisms possibly associated with Pt-resistance.

### 3.1. Mutational Landscape of TCGA-OV27 Cohort

**Figure 1:**
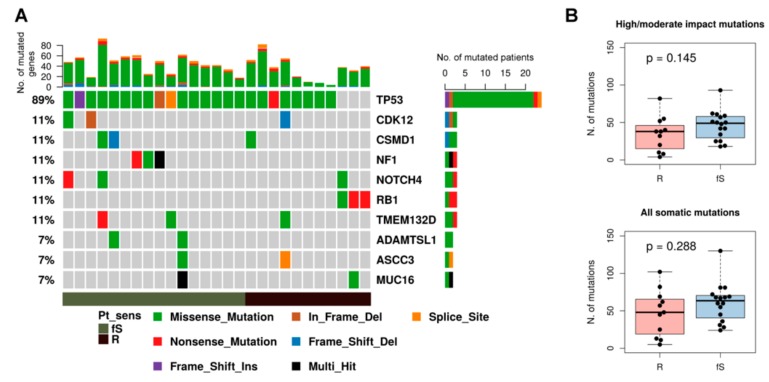
Mutational spectrum of TCGA-OV27 samples. (**A**) Oncoplot of the top-10 most frequently mutated genes in cytoreduction (R0) patients of the TCGA-OV27 dataset, grouped according to sensitivity class. Each column represents a sample and each row a different gene. Colored squares show mutated genes, while grey squares show no mutated genes. Different type of mutations are colored according to the variant type as indicated in the legend at the bottom. Genes annotated as “Multi_Hit” have more than one mutation in the same sample. The barplot at the top shows the number of mutated genes for each patient colored according to the mutation type. The barplot on the right reports the number of mutated patients for each gene, colored according to the mutation type. (**B**) Boxplot showing the tumor mutational load of R and fS samples, calculated both considering only mutations with high/moderate impact (upper panel) or all somatic mutations (lower panel). *P*-value was calculated by Wilcoxon rank-sum test.

To identify genomic features associated with early relapse we then analyzed mutational data of the selected cohort. Consistent with genome landscape studies of the whole TCGA-OV cohort and most recent studies on ovarian cancer [15,16], p53 is mutated in 89% of TCGA-OV27 patients (Figure 1). Among the prevalent mutated genes we found, as expected, *CDK12, NF1,* and *RB1*, together with *CSMD1*, *NOTCH4,* and *TMEM132D* genes, which seem to be a specific trait of this cohort. Considering genes mutated in at least three patients we did not observe any significantly unbalanced distribution of these predominantly mutated genes within R/fS classes (Figure 1A).

We compared the tumor mutational load in the two sensitivity classes considering either only mutations with high/moderate impact (Figure 1B upper panel) or all somatic mutations (Figure 1B lower panel); even if fS patients tend to have a slightly higher number of mutations, we did not detect significant differences between the two classes.

Overall, we identified in at least one sample 1115 variants with high/moderate impact affecting 1005 unique genes. To reduce the high inter-patient heterogeneity of mutational data we grouped genes into pathways and compared pathways mutated in R or fS patients. For this analysis we considered 50 gene sets from the ‘Hallmark’ collection of MSigDB database that summarize well-defined biological states or processes and ten canonical oncogenic pathways [13]. We called a pathway mutated if at least one of its genes was mutated. According to our analysis, none of the pathways tested was found to be significantly associated to sensitivity class, even at a less stringent nominal *p*-value < 0.05 (Tables S1 and S2).

### 3.2. Mutational Signatures of TCGA-OV27 Cohort

**Figure 2:**
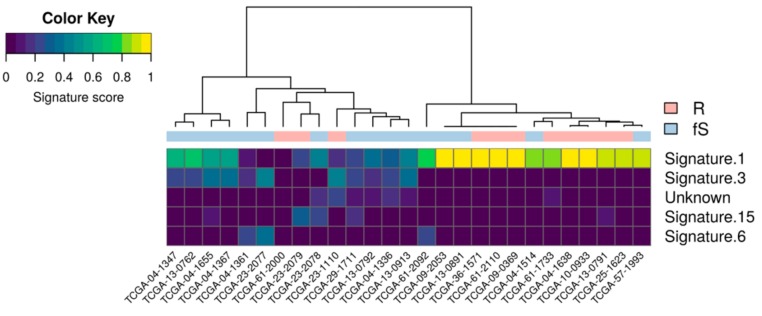
Mutational signatures in TCGA-OV27 cohort. Heatmap showing the contribution of the top-5 most represented COSMIC signatures in the mutational profiles of TCGA-OV27 samples.

Basing on the relative frequency of somatic base substitution events, 30 distinct mutational signatures, reflecting distinct mutational process associated with specific biological status and/or altered functions have been described (COSMIC; https://cancer.sanger.ac.uk/cosmic/signatures) [12]. These mutational signatures were analyzed in the TCGA-OV27 cohort considering all somatic variants independently of their functional consequences. A hierarchical clustering based on the scores of the five most represented mutational signatures identified two major clusters mainly driven by different contribution of Signature 1 (related to endogenous mutational processes) and Signature 3 (related to defective homologous repair of double-strand DNA break). Even if the association between these clusters and sensitivity was not significant (Fisher’s exact test *p*-value = 0.054), we observed a clear trend of enrichment of R patients in the cluster driven by Signature 1 and an enrichment of fS patients in the cluster driven by Signature 3 (Figure 2). The comparison of each signature’s score between the two classes is reported in Table S3 and a nominal *p*-value < 0.05 (Wilcoxon rank-sum test) was observed for Signature 3 only.

### 3.3. Genomic Instability and sCNA Landscape of TCGA-OV27 Cohort

On the basis of recent studies defining sCNA as the prevalent genomic alteration affecting HGSOC [17], we assessed whether R or fS patients of our selected cohort could be distinguished by specific sCNA.

**Figure 3:**
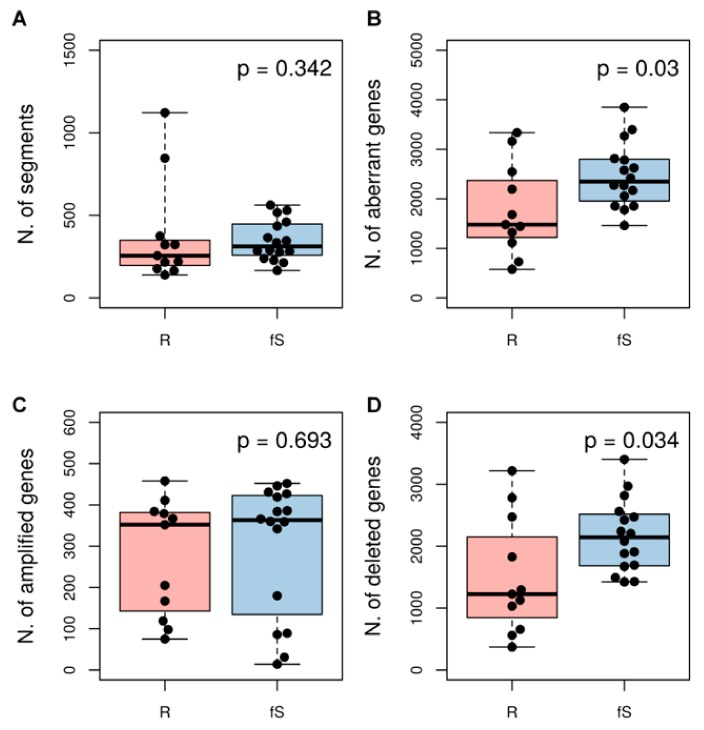
Association between genomic instability and sensitivity class in TCGA-OV27 dataset. As measure of genomic instability for each sample we considered: (**A**) the number of segments that represents the number of regions with different copy number levels within a genome; (**B**) the total number of amplified or deleted genes; the total number of amplified genes only (**C**) or deleted genes only (**D**). *P*-values are according to Wilcoxon rank-sum test.

We used sCNA data to obtain a measure of genomic instability for each patient, using different approaches all based on the GISTIC algorithm. We firstly considered the number of regions with different copy number (number of segments; Figure 3A), with the assumption that a higher number of segments should describe a more fragmented (instable) genome. We next considered within each sample either the total number of genes affected by sCNA (Figure 3B) or, separately, the total number of amplified or deleted genes (Figure 3C,D). Overall, we observed a trend for higher sCNA in fS compared to R patients. This trend was significant when we considered the number of genes affected by aberrations in general (Wilcoxon rank-sum test *p*-value = 0.03) and this difference was mainly driven by deletions (Wilcoxon rank-sum test *p*-value = 0.034).

**Figure 4:**
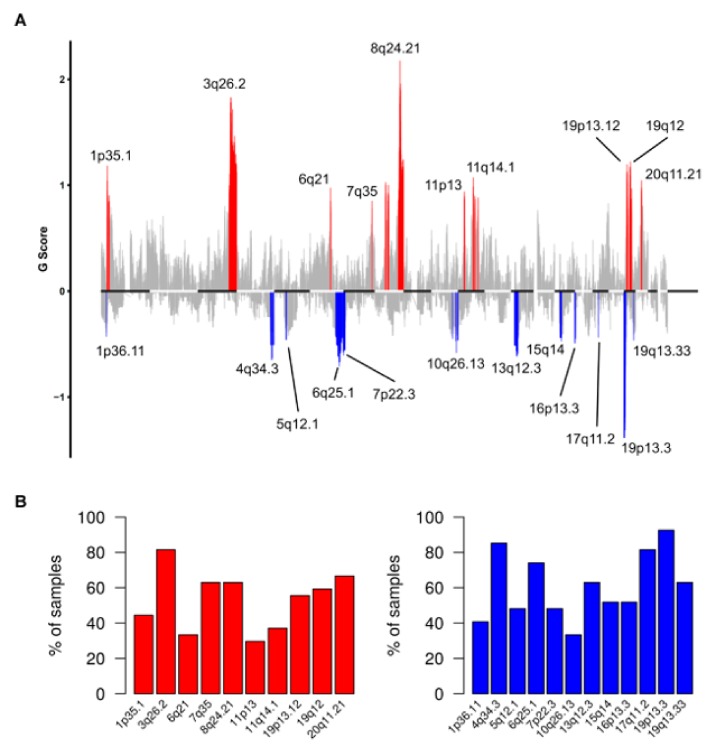
Recurrent somatic copy number alterations (sCNA) in R0 patients of TCGA-OV27 cohort. (**A**) Plot of G scores (defined as the amplitude of the copy number multiplied by its frequency across samples) calculated by Genomic Identification of Significant Targets in Cancer (GISTIC) for genomic regions recurrently amplified (red) or deleted (blue) in the TCGA-OV27 dataset, at an FDR < 0.1. (**B**) Barplot showing the frequency of samples positive for the recurrently amplified (left) or deleted (right) regions identified by GISTIC.

To identify recurrent sCNA we applied GISTIC to copy number data of the TCGA-OV27 cohort. We identified 10 regions significantly amplified and 12 regions significantly deleted across samples (Figure 4A). All sCNA events, as well as detail in category, chromosome location genes in the region and cytobands are in Table S4.

The frequency plot distribution of sCNA detected in the TCGA-OV27 cohort (Figure 4B), shows that 3q26.2 gain, 17q11.2, 19p13.3, and 4q34.3 loss were the most frequently altered regions (>80% of patients). The frequency of samples positive for the recurrently amplified or deleted regions in the two subgroups (R and fS) of patients is showed in Figure S2. However, when we compared the frequency of the recurrent sCNA identified by GISTIC between the two sensitivity classes, no significant association to Pt-sensitivity was observed (Table S5).

We repeated the analysis of sCNA at the gene-level, considering 23110 amplified or deleted genes. Due to the low number of samples available for each class, no significant findings were detected after multiple-testing correction, while we detected 1270 genes more frequently altered at a nominal *p*-value < 0.05 (Fisher’s exact test) in R or fS patients (166 amplified and 1104 genes deleted, Table S6). Considering the explorative nature of this pilot study, we further explored the genes list being aware of the limitations associated with analysis of small group of patients and the high risk of detecting false positive hits (see Materials and Methods, Section 2.5 for power calculation of the present study). Among these genes we observed that amplifications were more frequently detected in R rather than in fS group (median percentage of patients with amplified genes: 57% in R vs 12% in fS; Range: 36–82% in R group, 0–37% in fS group). On the other side deletions were more frequently detected in fS patients (median percentage of patients with deleted genes: 15% in R vs 62% in fS group; Range: 0–63% in R group, 19–87% in fS group).

### 3.4. Association between sCNA and Altered Gene/Pathways Expression in Pt-Sensitivity Classes

We investigated the relationships between sCNA, alteration of genes’ expression and relevant functional pathways possibly affected by these alterations.

**Figure 5:**
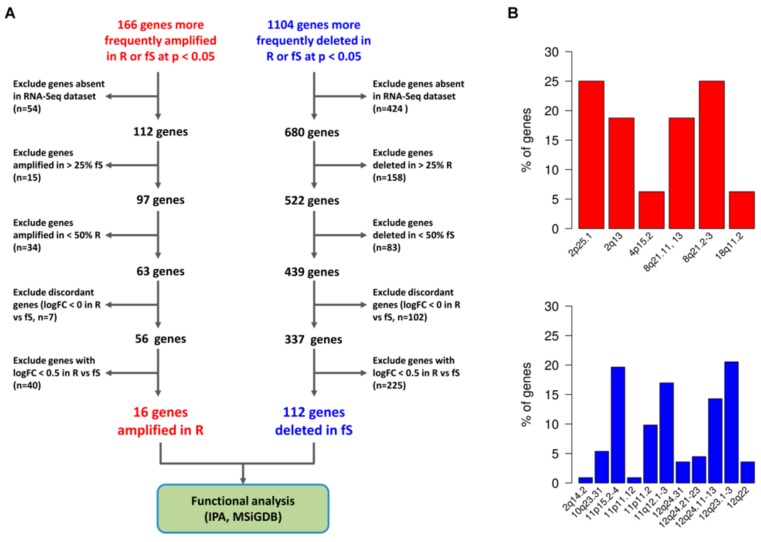
Association between sCNA and altered gene expression in Pt-sensitivity classes. (**A**) Selection of significant focal sCNA with concordant alteration of gene expression. Gene expression was assessed by RNA-sequencing (RNASeq) data, and for each altered gene, the logFC expression ratio of R vs fS patients was calculated. The workflow guiding selection of both amplified and deleted genes with concordant expression is shown. (**B**) Cytobands associated with significant sCNA and altered gene expression. In the plot are reported the cytobands affected by significant amplification (upper panel, red bars) and deletions (lower panel, blue bars). For each type of alteration, the relative frequency of each cytoband affected is shown.

The complete decision tree for gene selection is shown in Figure 5A. We first removed genes that were not assessed by RNA-Seq from the list of 1270 genes significantly amplified or deleted in R or fS patients. We next filtered this list according to the observed relative frequency of amplification or deletion for each sensitivity class. Finally, we removed genes whose log2 fold change (FC) was not compatible with its copy number status (e.g., a gene preferentially amplified but down-regulated in R group) and among the concordant genes we selected those with a log2 FC of at least 0.5 between R and fS patients. The final gene list included 128 genes (Table S7), consisting of 16 genes preferentially amplified and up-regulated in R group and 112 genes more frequently deleted and down-regulated in fS patients. The relative frequency of associated altered cytobands is reported in Figure 5B. Interestingly, these cytobands were not included among the significantly recurrent aberrant regions identified by GISTIC.

**Figure 6:**
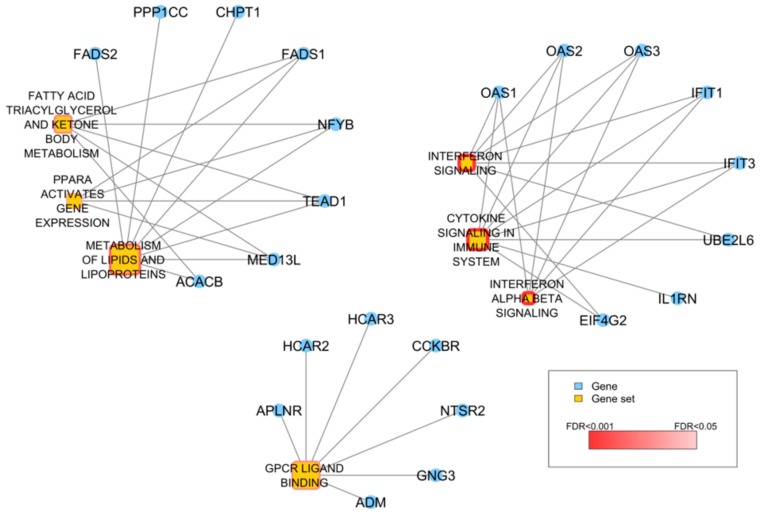
Over-representation analysis of the 128 genes with concordant sCNA and expression. Network showing the 7 Reactome gene sets significantly over-represented in the list of 128 genes. Yellow nodes represent gene sets and the size of the node is proportional to the number of genes catalogued in the gene set. The significance of the over-representation is represented by a dark-to-light red color scale. Blue nodes represent genes and are connected to a gene set if they are among its gene members.

To map the 128 altered genes into known functional pathways we firstly assessed over-representation of Reactome canonical pathways included in the C2 gene set collection of MSiGDB. Seven gene sets, related to interferon (IFN) and cytokine signaling, fatty acid and lipid metabolism, were found significantly over-represented (FDR < 0.05) and 23 out of the 128 genes overlapped with at least one of them (Figure 6).

Then, to examine biological relationship and investigate functional effects related to sCNA of these 128 genes, we run Ingenuity Pathway Analysis. Canonical pathways analysis confirmed a significant modulation in IFN signaling, mostly related to *IFIT* and *OAS2* genes deletion, and fatty acid metabolism dependent on alterations in desaturase genes (FADS1 and FADS2). We also observed significant modulation of G-alpha proteins signaling pathways (Table 2). The most significant Regulatory Networks affected by the altered gene expression are listed in Table 3.

**Figure 7:**
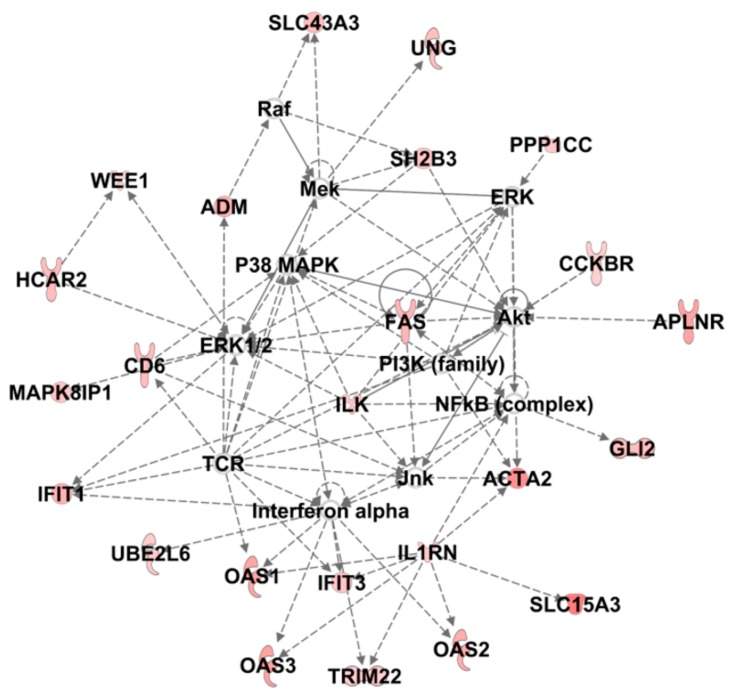
IPA analysis of the 128 significantly altered genes. The top-scoring regulatory network built from Core Analysis and named Dermatological Diseases and Conditions, Organismal Injury and Abnormalities, Immunological Disease is shown. Colored nodes are the genes of the dataset participating to the network.

The top-scoring Network (Dermatological Diseases and Conditions, Organismal Injury and Abnormalities, Immunological Disease) whose graphical representation is reported in Figure 7, shows a number of molecular relationships centered on interferon, in accordance with previously described pathway analysis. Interestingly, among the top regulatory networks we also identified a number of networks whose functions are mainly related to cell growth and proliferation and cell development. Accordingly, Cellular Development, Cell Morphology, Cellular Growth and Proliferation were the most significantly modulated Molecular Cell Function (Table S8), while Invasion, Migration and Cell Movement were the only Disease and Functions significantly predicted to be increased in R patients (Table S9).

## 4. Discussion

The relationship between post-operative residual tumor burden and clinical outcome is consolidated for ovarian cancer [2,18]. Consequently, the concept of optimal cytoreduction is evolving along time and, although the metric for optimal debulking is still defined as tumor nodules not greater than 1 cm, the literature and meta-data analysis clearly show that R0 patients, those with no residual tumor after primary surgery, have the best overall outcome. Nevertheless, some of those R0 patients with an expected favorable clinical outcome still experience early disease relapse, due to intrinsic molecular characteristics of their tumors. We focused our attention on this small, poorly characterized subgroup of patients with an unexpectedly unfavorable prognosis (R patients; PFS < 12 months from surgery). To possibly identify molecular traits associated with Pt-resistant disease in R0 patients we analyzed their genomic portrait in comparison to those of R0 patients with good prognosis (fS patients; PFS > 24 months from surgery).

The approach to compare the gene expression of two series of patients with marked opposite outcomes, with the assumption that this selection may enhance discovering relevant molecular pathways associated to sensitivity to pt- or cetuximab/pt-based treatment was previously applied with some success to small cohorts of gastric [19] and head and neck cancers [20]. Comparison of transcriptomes in the two R0 HGSOC Pt-sensitivity classes, showed in R patients modifications that could be commonly referred to an increased aggressiveness of tumors but were not directly suggestive of specific actionable alterations. Similarly, the mutational profile of the R0 cohort did not substantially differ from the overall TCGA study population, where, apart the prevalent p53 mutation, few other genes were commonly mutated and in a small fraction of patients, thus confirming the high mutational heterogeneity of this tumor type. Also, none of these prevalently mutated genes was significantly associated with Pt-sensitivity classes. Interestingly, from the overall analysis of the somatic mutational status we did not observe any enrichment for *BRCA1/2* mutations in the fS group and not all fS patients were characterized by mutational Signature 3 related to defective Homologous Repair of double-strand DNA breaks. These findings are substantially in agreement with recent data obtained on long term survivor ovarian cancer patients [15] where the authors propose that the BRCA-associated signature alone could not be prognostic of Pt-sensitivity in HGSOC. Nevertheless, the analysis of genomic instability in our R0 cohort disclosed a higher number of sCNA in fS patients as compared to R ones. Since genomic instability can be attributed to defects in HR pathway [21] these data are in accordance with the observed enrichment of fS patient in the cluster characterized by mutational Signature 3 and overall support their Pt-sensitivity. This trend appeared to be mainly driven by deletions rather than amplification, in accordance with data obtained from focal (gene-level) sCNA analysis, which described deletions as the prominent event distinctive of the fS group. At the same time these observations confirm that the maintenance of potential oncogenic pro-survival functions is a requirement for Pt-resistance and, concordantly, their inactivation might be an opportunity to overcome Pt-resistance. Noteworthy, the sCNA profile of the R0, fS patients of the TCGA-OV27 cohort appeared to be different from the HR-deficient TCGA-OV cohort [5], possibly because of the specific molecular setting of R0 tumors, which have been shown to be intrinsically different from those ovarian tumors more massively diffused in the peritoneal cavity and less-likely to be completely removed at primary surgery [22].

sCNA are known drivers of HGSOC development and progression [17]. Interestingly, in a recent paper studying the spatial and temporal heterogeneity of HGSOC [23], the authors suggest that in this tumor type the relapsed disease is mostly related to the emergence of pre-existing rather than de-novo clones and observe that sCNA maintain a low level of intra-patient heterogeneity. Therefore, sCNA analysis promises to be an effective strategy to identify cancer-causing genes, which could be used for treatment decisions. Accordingly, Cyclin E (*CCNE1*) amplification is a known trait in ovarian cancers with intact HR. It occurs in around 20% of all HGSOC and since it is mutually exclusive with *BRCA1/2* mutation [24], patients harboring *CCNE1* amplification will not benefit from *PARP*i treatment and will likely be less responsive to Pt treatment [5,17]. Nevertheless, these finding provided the rationale for the development of therapeutic approach that specifically exploit the tumor dependence upon *CCNE1* amplification, for instance by targeting *CDK2* and *AKT* activities [25].

*CCNE1* locates on chr19q12 cytoband, which we identified to be amplified in the whole TCGA-OV27 cohort. However, possibly due to the small number of samples analyzed, we did not identify any significant enrichment of 19q12 amplification in the R subgroup of patients and, concordantly, we did not have any evidence about significant enrichment of *CCNE1* amplification in the same subgroup when we performed the analysis at gene-level. Of note, none of the cytobands identified as significantly altered in both R and fS class of R0 patients at gene level is comprised in the recurrent chromosomal aberrations identified and only 5 genes (*FAS, HEY1, SH2B3, TBX3, USP44*) were included in the COSMIC (Catalogue of Somatic Mutations in Cancer) Cancer Gene Census list, suggesting that new information can be acquired with this approach.

Among these newly identified genes, we found *HEY1*, to be amplified in the R group. Interestingly, *HEY1* is a downstream mediator of Notch-dependent signals [26], it has a putative role as oncogene (COSMIC) and its expression was recently associated with an EMT phenotype, increased invasion and cell migration as well as Pt resistance in head and neck cancers [27]. These observations are in agreement with IPA describing a predicted increase in functions (cell growth and proliferation and cell development) overall suggestive of a stemness program. Also, Notch1 signaling pathway has been described to contribute to chemoresistance in ovarian cancer [28], it is a key for maintenance of cancer stem cell in ovarian cancer [29] and the development of new treatment strategies targeting these pathways to control stem-cell replication is a current active field of research.

Pt-resistance is recognized to be a multifactorial event and the search for determinants (genes) guiding response to Pt treatments still continues to be a key issue in ovarian cancer translational research. In this context, it has been proven the involvement of aberrant DNA methylation and modification of histone marks [17,30] in the development of Pt-resistance and a number of synthetically lethal approaches are under investigation, with cell cycle check points (CHK1, Wee1, DNA-PK) and related cyclins inhibitors being among the most promising (see [31,32] for an overview).

To our knowledge, this is the first study of R0 HGSOC that specifically investigates sensitivity to Pt-based therapy by transcriptomics and genomics analyses and biology behind. We are aware that our study has to be considered explorative. The major limit rests in the small number of samples included in the TCGA-OV27 cohort, which account for about 6% of the entire TCGA-OV cohort. This limitation is inherently related to the reduced number of ovarian cancer patients having these clinical characteristics and with full molecular data available. The statistical power of our analyses is constrained to 2.4% by the sample size of the TCGA-OV27 cohort, which should triplicate to endow a statistical significance. Our results should be interpreted with caution. Nevertheless, the overall data presented here, based on tumors with marked opposite treatment outcomes, are suggestive of a specific Pt-resistance molecular trait driven by sCNA, and these observations deserve to be further explored in wider cohort of patients with selected clinical characteristics. If verified and upon appropriate independent validation, it could possibly drive toward the development of a new tool based on the sCNA pattern, which may help clinicians in defining sensitivity to Pt treatment.

## Figures and Tables

**Table 1:** Clinical characteristics of The Cancer Genome Atlas (TCGA) ovarian serous cystadenocarcinoma (TCGA-OV) 27 cohort.

	Total (n = 27)	R (n = 11)	Fs (n = 16)
**Stage**			
III	23	9	14
IV	4	2	2
**Grading**			
G2	3	0	3
G3	23	10	13
NA	1	1	0
**Relapse**			
yes	18	11	7
no	9	0	9

R = Resistant; fS = frankly Sensitive.

**Table 2:** Canonical Pathways identified by Ingenuity^®^ Pathway Analysis (IPA).

Ingenuity Canonical Pathways	-Log(*p*-Value)	Genes
Interferon signaling	2.87	*OAS1, IFIT1, IFIT3*
Oleate biosynthesis II (animals)	2.8	*FADS1, FADS2*
Graft-versus-host Disease signaling	2.62	*IL1RN, IL36RN, FAS*
Gαs signaling	2.4	*CNGB3, GNG3, HCAR3, HCAR2*
γ-linolenate biosynthesis II (animals)	2.33	FADS1, FADS2
Gαi signaling	2.15	*APLNR, GNG3, HCAR2, CHRM4*

**Table 3:** Top regulatory networks identified by IPA. Genes detected in the TCGA-OV27 cohort are shown in bold.

Top Diseases and Functions	Molecules in Network	Score	Focus Molecules
**Dermatological diseases and conditions, Organismal injury and abnormalities, immunological disease**	**ACTA2**, **ADM, APLNR**, Akt, **CCKBR**, **CD6,** ERK, ERK1/2, **FAS**, **GLI2, HCAR2**, **IFIT1, IFIT3, IL1RN, ILK,** Interferon alpha, Jnk, **MAPK8IP1**, Mek, NFkB (complex), **OAS1, OAS2, OAS3**, P38 MAPK, PI3K (family), **PPP1CC**, Raf, SH2B3, **SLC15A3, SLC43A3**, TCR, **TRIM22, UBE2L6, UNG, WEE1**	44	24
**Gastrointestinal disease, organismal injury and abnormalities, cell death and survival**	**ADM, AHNAK,** ATG7, C3, CFTR, CLDN7, CST5, CTSS, **DENND5**A, DUSP10, **FADS1, FADS2**, HAS1, **HCAR3**, HLA-B, IFNGR1, IL13, IL1B, **IL36RN**, LBP, MAFF, **MS4A4A**, NFKBIE, **NRIP3, PQLC3, SEL1L3, SLC43A3,** SMARCA4, **STK33**, **STMN2, SYT7**, TNF, TP63, **TUB, WWP1**	27	17
**Gene expression, cell cycle, cellular growth and proliferation**	ACAD10, BAG1, CBS/CBSL, CCNB2, **CDK17, CORO1C,** CTR9, **DCHS1**, ESR1, **FGD6, GREB1**, HAUS8, HDAC1, HLTF, LTB, **NEDD1, NFYB**, NR1D1, **NR2C1**, NR3C1, NUPR1, PLK1, PRMT6, **SCUBE2,** SMARCE1, SMYD2, SMYD3, SP1, **TBX3, TEAD1**, TFAP2C, TNFAIP6, **TRAFD1,** YWHAG, estrogen receptor	19	13
**Cellular development, cellular growth and proliferation, cell cycle**	CCND1, CDCA2, CDK5, CHD7, **CMKLR1,** Ctbp, **DRAM1, E2F5**, ERBB2, **GCN1**, **HCAR1, HEY1**, JAG1, LINC-ROR, **LIPF**, MAFB, **MED13L**, NOTCH2, NOTCH4, **NUAK1,** NUMB, OIP5-AS1, PCLAF, PPARGC1A, RFC1, **RMST**, RUNX3, SLC16A1, SMTN, SOX2, SUV39H1, **TMEM119, TMPO**, TP53, let-7a-5p	17	12
**Gene expression, cell signaling, cellular development**	26s Proteasome, **ACACB, ACTA2**, AR, **ASCL1, ATP6V0D2,** BAG1, CD55, CDK5, CDKN1C, **CHRM4, CHST1**, CKAP4, DAB2, DLL4, FSH, GBP1, H2AFY, HES1, **HRK**, IER3, **LYVE1,** Lh, MED12, MTOR, NOTCH1, PGR, PRKD1, PRKD2, SMARCE1, **SMTNL1, SSH1**, TOP1, **TP53I11**, YWHAB	17	12
**DNA replication, recombination, and repair, cell morphology, cellular function and maintenance**	ACTG1, **CHPT1**, CLOCK, DDX11, DDX5, **DTX4, EIF4G2**, EP300, **FEN1**, GATA1, **HBB**, HNRNPC, HNRNPD, HNRNPU, HUS1, MAX, MYB, **OAS3**, OTUB1, **PARPBP,** PCLAF, PCNA, RAD51, RAD9A, RFC1, RHOA, Rnr, SATB1, **TMEM241**, TP53BP1, **TRPV4**, **USP44, UTP20**, XRN2, YBX1	15	11

## Supplementary Materials

The following are available online at https://www.mdpi.com/2073-4425/10/9/678/s1, Figure S1: Enrichment plots of hallmark gene sets significantly enriched at an FDR < 0.01. Gene sets were tested against the list of genes ranked according to their differential expression significance and fold change, from the most up-regulated to the most down-regulated in R versus fS. The Gene Ranks column shows the location of the genes of each gene set along the pre-ranked gene list. NES: normalized enrichment score; padj: adjusted *p*-value (FDR). Figure S2: Barplot showing for each sensitivity class the frequency of samples positive for the recurrently amplified (left panel) or deleted (right panel) regions identified by GISTIC. Table S1. Association between mutated hallmark gene sets and sensitivity class. *P*-value by Fisher’s exact test. FDR: false discovery rate. Table S2. Association between mutated oncogenic pathways and sensitivity class. *P*-value by Fisher’s exact test. FDR: false discovery rate. Table S3. Association between mutational signatures scores of the top-5 most represented COSMIC signatures and sensitivity class. *P*-value by Wilcoxon rank-sum test. FDR: false discovery rate. Table S4. Recurrently amplified or deleted regions in the TCGA-OV27 dataset identified by GISTIC. Table S5. Association of recurrently amplified or deleted regions identified by GISTIC and sensitivity class. *P*-value by Fisher’s exact test. FDR: false discovery rate. Table S6. Association of copy number status at the gene level and sensitivity class. *P*-value by Fisher’s exact test. Table S7. List of the 128 genes selected after integration of sCNA and RNA-Seq data. Table S8. IPA on 128 genes list. Molecular cell functions significantly modulated Table S9. Prediction of Disease and Function activation state by IPA on 128 genes list.
