# Supplementary figures and images for: Focal Recurrent Copy Number Alterations Characterize Disease Relapse in High Grade Serous Ovarian Cancer Patients with Good Clinical Prognosis: A Pilot Study
Source: Genes (Basel). 2019 Sep 5;10(9):678. doi: 10.3390/genes10090678 (PMC6770978; doi:10.3390/genes10090678)

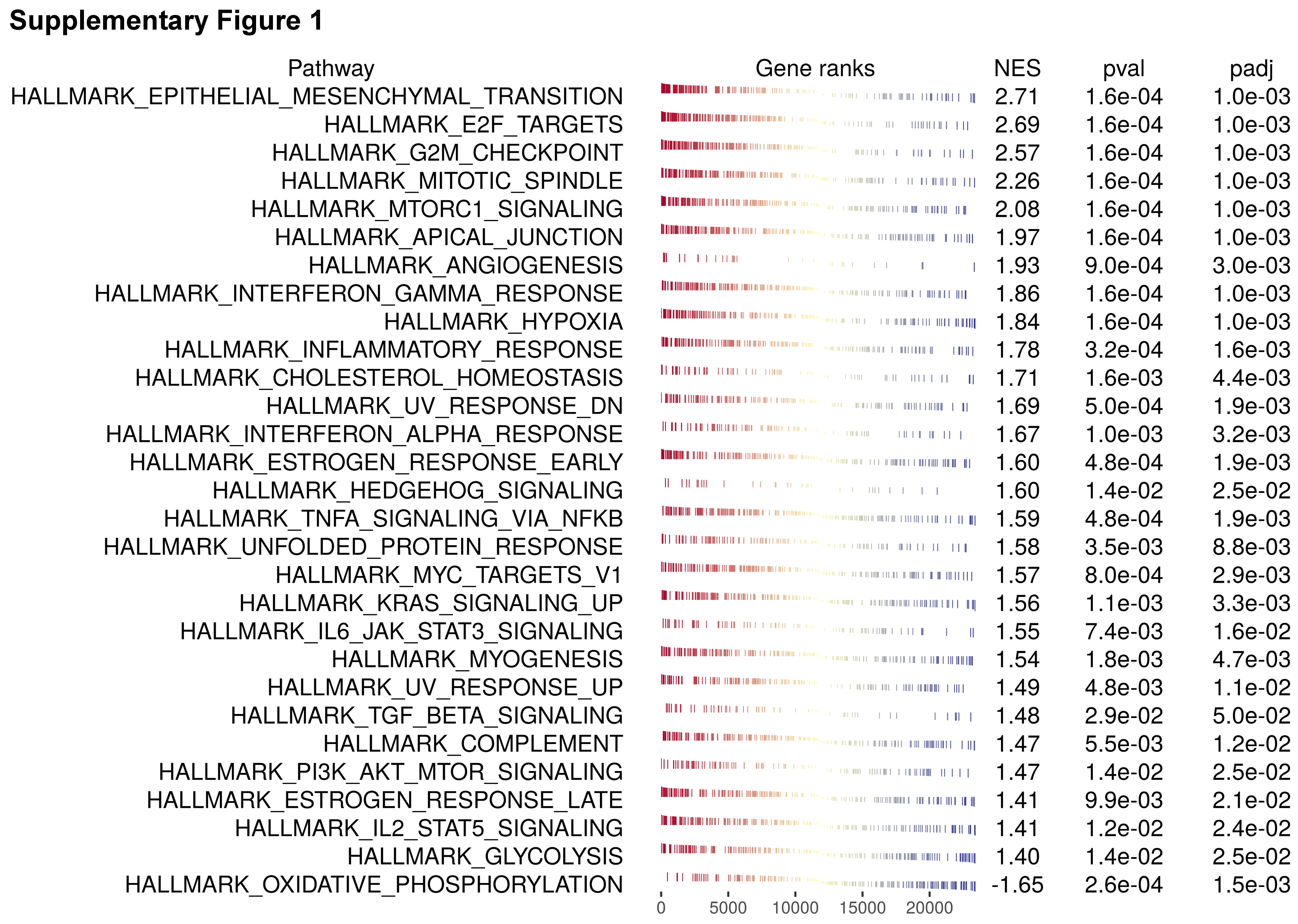

Supplement: Supplementary file 1 [file genes-10-00678-s001.zip › Supplementary_Figure_1.tif]

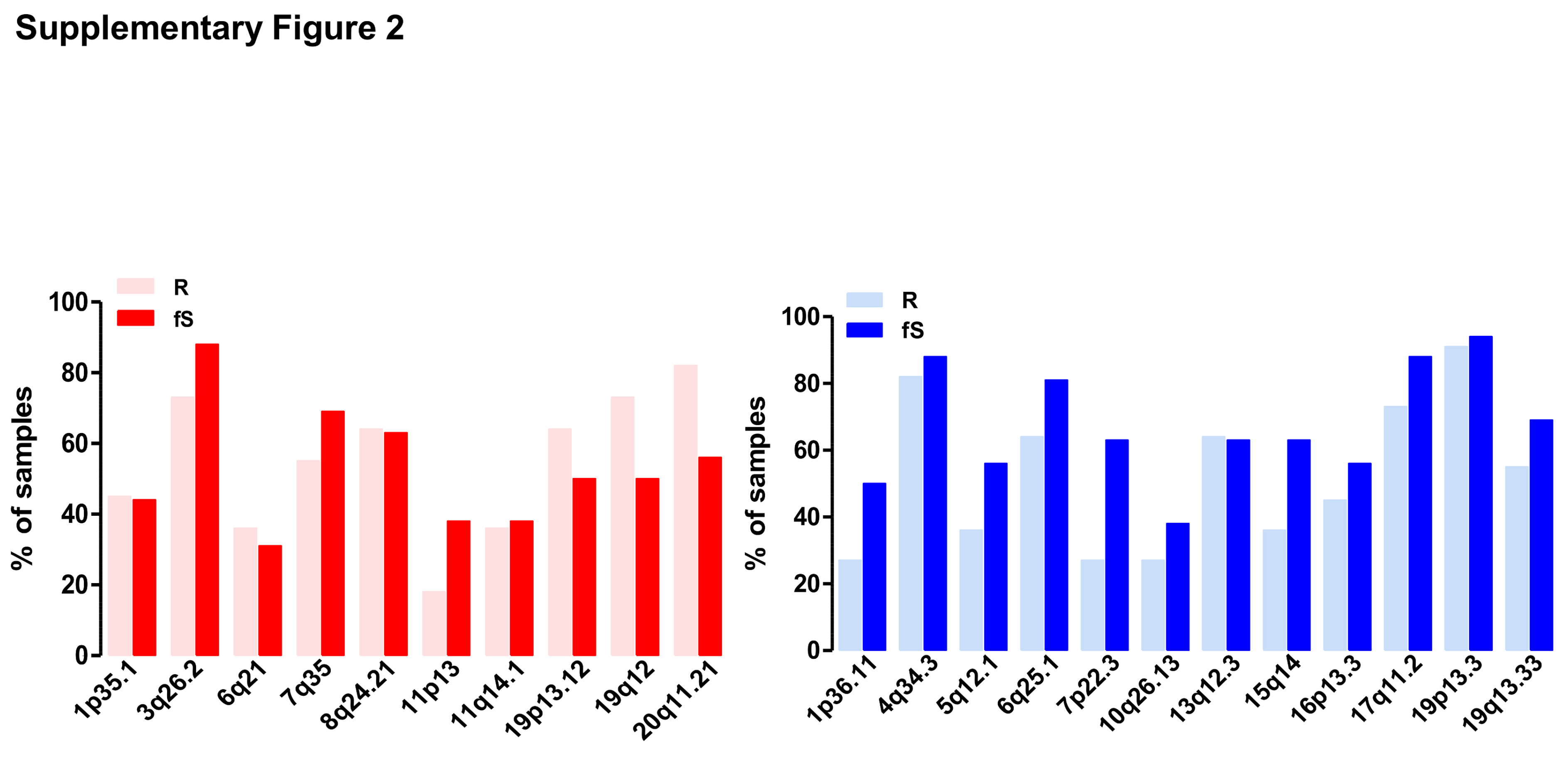

Supplement: Supplementary file 1 [file genes-10-00678-s001.zip › Supplementary_Figure_2.tif]
